# Supplementary material for: The value of TI-RADS combined with superb microvascular imaging in distinguishing thyroid nodules: A protocol for systematic review and meta-analysis
Source: PLoS One. 2021 Jan 7;16(1):e0245035. doi: 10.1371/journal.pone.0245035 (PMC7790367; doi:10.1371/journal.pone.0245035)
Supplement: S1 Checklist — (DOC) [file pone.0245035.s001.doc]

**PRISMA-P (Preferred Reporting Items for Systematic review and Meta-Analysis Protocols) 2015 checklist: recommended items to address in a systematic review protocol***

| Section and topic | Item No | Checklist item | Reported on Page # |
| --- | --- | --- | --- |
| ADMINISTRATIVE INFORMATION | | |  |
| Title: |  |  |  |
| Identification | 1a | The value of TI-RADS combined with superb microvascular imagine in distinguishing thyroid nodules: A protocol for systematic review and meta-analysis | 1 |
| Update | 1b | NA |  |
| Registration | 2 | INPLASY202070113 | 2 |
| Authors: |  |  |  |
| Contact | 3a | *Corresponding author: Congliang Tian, fytgzy@163.com, Master of Medicine, 8618098824068.  *Corresponding author: Lin Zhong, 365213427@qq.com, Master of Medicine, 8618098877106.  Address: No.222 Zhongshan Road, Xigang District, Dalian City, Liaoning Province, China. | 1 |
| Contributions | 3b | Conceptualization: Cong Wang, Mingxin Lin.  Data curation: Cong Wang, Congliang Tian, Lin Zhong.  Methodology: Cong Wang, Mingxin Lin.  Writing – original draft: Congliang Tian, Mingxin Lin.  Writing – review & editing: Cong Wang, Lin Zhong. | 6 |
| Amendments | 4 | NA |  |
| Support: |  |  |  |
| Sources | 5a | This study is supported by Liaoning Natural Science Foundation Project (20170540256). | 1 |
| Sponsor | 5b | Xiukun Hou. | 1 |
| Role of sponsor or funder | 5c | Provider just financially supports this study, but does not involve all sections of this study, and does not have conflicts interest related to this study | 1 |
| INTRODUCTION | | |  |
| Rationale | 6 | Previous studies have shown that SMI can detect the blood flow signals of neovascularization in tumor and increased the sensitivity for detecting thyroid cancer.[9-12] However, the results of these studies have been contradictory and the sample sizes were not enough. | 3 |
| Objectives | 7 | Therefore, the present meta-analysis aimed at evaluating the value of TI-RADS combined with SMI in distinguishing benign and malignant thyroid nodules. | 3 |
| METHODS | | |  |
| Eligibility criteria | 8 | Type of study. This study will only include high quality clinical cohort or case control studies.  Type of patients. The patients should be those who had undergone thyroid nodule.  Intervention and comparison. Thyroid nodules of all patients were assessed with single TI-RADS and TI-RADS combined with SMI.  Type of outcomes.  The primary outcomes include area under the curve (AUC) of summary receiver operating characteristic (SROC), through which thyroid nodules was assessed by means of both TI-RADS and TI-RADS combined with SMI. | 4 |
| Information sources | 9 | PubMed, Web of Science, Cochrane Library, and Chinese biomedical databases will be searched from their inceptions to the June 31, 2020, without language restrictions. | 4 |
| Search strategy | 10 | The following keywords and MeSH terms were used: ["thyroid cancer" or "thyroid neoplasm" or "thyroid tumor" or "thyroid nodule "] and [“superb microvascular imaging”]. We also performed a manual search to find other potential articles. | 4 |
| Study records: |  |  |  |
| Data management | 11a | Two authors will independently select the trials according to the inclusion criteria, and import into  Endnote X9. | 3 |
| Selection process | 11b | Remove duplicated or ineligible studies. Screen the titles, abstracts, and full texts of all literature to identify eligible studies. | 3 |
| Data collection process | 11c | All essential data will be extracted using previously created data collection sheet by 2 independent authors. Discrepancies in data collection between 2 authors will be settled down through discussion with the help of another author. | 4 |
| Data items | 12 | the first author's surname, publication year, language of publication, study design, sample size, number of lesions, source of the subjects, "gold standard," and diagnostic accuracy. The true positives (TP), true negatives (TN), false positives (FP), and false negatives (FN) in the fourfold (2 x 2) tables were also collected | 4 |
| Outcomes and prioritization | 13 | The summary receiver operating characteristic (SROC) curve and corresponding area under the curve (AUC) will be obtained. | 4 |
| Risk of bias in individual studies | 14 | Methodological quality was independently assessed by two researchers based on the quality assessment of studies of diagnostic accuracy studies (QUADAS) tool | 4 |
| Data synthesis | 15a | We will calculate the pooled summary statistics for sensitivity (Sen), specificity (Spe) with their 95%confidence intervals (CIs). The summary receiver operating characteristic (SROC) curve and corresponding area under the curve (AUC) will be obtained. We will compare the two AUC areas of single TI-RADS and TI-RADS combined with SMI. | 4 |
|  | 15b | The Cochran’s Q-statistic and I2 test will be used to evaluate potential heterogeneity between studies.[13] If the Q-test shows a P<0.05 or I2 test exhibits >50%, indicating significant heterogeneity, and the random effect model will be employed or if heterogeneity is not significant, the fixed-effects model was used | 5 |
|  | 15c | Sensitivity analysis will be performed to evaluate the influence of a single study on the overall estimate. | 5 |
|  | 15d | If it is possible, we will perform meta-analysis to analyze the pooled outcome data when acceptable homogeneity has been identified. Otherwise, we will conduct subgroup analysis to investigate potential causes for substantial heterogeneity among eligible studies. | 5 |
| Meta-bias(es) | 16 | We will use Begger’s funnel plots and Egger’s linear regression test to investigate publication bias. | 5 |
| Confidence in cumulative evidence | 17 | we will perform a systematic review to summarize high-quality studies and to provide evidence on the evidence-based medical support for clinical practice. | 6 |

*** It is strongly recommended that this checklist be read in conjunction with the PRISMA-P Explanation and Elaboration (cite when available) for important clarification on the items. Amendments to a review protocol should be tracked and dated. The copyright for PRISMA-P (including checklist) is held by the PRISMA-P Group and is distributed under a Creative Commons Attribution Licence 4.0.**

*From: Shamseer L, Moher D, Clarke M, Ghersi D, Liberati A, Petticrew M, Shekelle P, Stewart L, PRISMA-P Group. Preferred reporting items for systematic review and meta-analysis protocols (PRISMA-P) 2015: elaboration and explanation. BMJ. 2015 Jan 2;349(jan02 1):g7647.*
