# Supplementary material for: The value of TI-RADS combined with superb microvascular imaging in distinguishing thyroid nodules: A protocol for systematic review and meta-analysis
Source: PLoS One. 2021 Jan 7;16(1):e0245035. doi: 10.1371/journal.pone.0245035 (PMC7790367; doi:10.1371/journal.pone.0245035)
Supplement: Attachment — Submitted filename: Response to Reviewers.docx [file pone.0245035.s003.docx]

1. Please ensure that your manuscript meets PLOS ONE's style requirements, including those for file naming. The PLOS ONE style templates can be found at

https://journals.plos.org/plosone/s/file?id=wjVg/PLOSOne_formatting_sample_main_body.pdf and

https://journals.plos.org/plosone/s/file?id=ba62/PLOSOne_formatting_sample_title_authors_affiliations.pdf

**Answer：ok**

2.Thank you for stating the following financial disclosure:

[Initials of the authors who received each award. The funders will not have a role in study design, data collection and analysis, decision to publish, or preparation of the manuscript.].

At this time, please address the following queries:

Please clarify the sources of funding (financial or material support) for your study. List the grants or organizations that supported your study, including funding received from your institution.

State what role the funders took in the study. If the funders had no role in your study, please state: “The funders had no role in study design, data collection and analysis, decision to publish, or preparation of the manuscript.”

If any authors received a salary from any of your funders, please state which authors and which funders.

If you did not receive any funding for this study, please state: “The authors received no specific funding for this work.”

Please include your amended statements within your cover letter; we will change the online submission form on your behalf.

**Answer:Line23-25.**

3. We note that you have stated that you will provide repository information for your data at acceptance. Should your manuscript be accepted for publication, we will hold it until you provide the relevant accession numbers or DOIs necessary to access your data. If you wish to make changes to your Data Availability statement, please describe these changes in your cover letter and we will update your Data Availability statement to reflect the information you provide.

**Answer**:OK.

4. PLOS requires an ORCID iD for the corresponding author in Editorial Manager on papers submitted after December 6th, 2016. Please ensure that you have an ORCID iD and that it is validated in Editorial Manager. To do this, go to ‘Update my Information’ (in the upper left-hand corner of the main menu), and click on the Fetch/Validate link next to the ORCID field. This will take you to the ORCID site and allow you to create a new iD or authenticate a pre-existing iD in Editorial Manager. Please see the following video for instructions on linking an ORCID iD to your Editorial Manager account: https://www.youtube.com/watch?v=_xcclfuvtxQ

**Answer**:OK.

5. Please amend either the abstract on the online submission form (via Edit Submission) or the abstract in the manuscript so that they are identical.

**Answer**:OK.

6. Please include captions for your Supporting Information files at the end of your manuscript, and update any in-text citations to match accordingly. Please see our Supporting Information guidelines for more information: http://journals.plos.org/plosone/s/supporting-information.

**Answer**:OK.

Additional Editor Comments (if provided):

The authors should use Google Scholar as a search engine or to explain why particular search engines were chosen.

**Answer**:OK.

The headline refers to both systematic review and meta-analysis while the abstract refers to systematic review alone. As this are two distinct entities, the authors should clarify which type of work is about to be done.

**Answer**:This is only a ***protocol*** for systematic review and meta-analysis.

The authors must adhere to the PRISMA protocol for systematic review and meta-analysis. This should be stated and carries out throughout the manuscript.

**Answer**:OK.

The authors should explain better current knowledge on regular Doppler and why it was not adopted for ACR TIRADS, and then address differences of regular Doppler and SMI

The type of studies is not well defined. Please adhere to the PRISMA protocols

**Answer**:Line61-67.

How can the authors define 'without language restriction'? How do the plan to overcome this barrier in order to fully understand the articles?

**Answer**:I have deleted 'without language restriction'.

Why does criteria 1 in table 1 needed? Please explain

**Answer**: We know the PICOS principle. Criteria 1 corresponds P(participant). Of course, the absence of criteria1 has little effect on the retrieval results because Criteria 2.

Discussion does contain a 'will be' which will address the study's result. Is it just the opening statement?

**Answer**: Make you laugh. This is a protocol in the future tense.
